# Reliability, accuracy, and minimal detectable difference of a mixed concept marker set for finger kinematic evaluation^☆^

**DOI:** 10.1016/j.heliyon.2023.e21608

**Published:** 2023-10-31

**Authors:** Manuela Paulina Trejo Ramirez, Neil Evans, Matthew Venus, Joseph Hardwicke, Michael Chappell

**Affiliations:** a School of Engineering, University of Warwick, Coventry, United Kingdom of Great Britain, And Northern Ireland, UK; b Institute of Applied and Translation Technolgies in Surgery, University Hospitals Coventry & Warwickshire NHS Trust, Coventry, United Kingdom of Great Britain, And Northern Ireland, UK

## Abstract

The study of finger biomechanics requires special tools for accurately recording finger joint data. A marker set to evaluate finger postures during activities of daily living is needed to understand finger biomechanics in order to improve prosthesis design and clinical interventions. The purpose of this study was to evaluate the reliability of a proposed hand marker set (the Warwick marker set) to capture finger kinematics using motion capture. The marker set consisted of the application of two and three marker clusters to the fingers of twelve participants who participated in the tests across two sessions. Calibration markers were applied using a custom palpation technique. Each participant performed a series of range of motion movements and held a set of objects. Intra and inter-session reliability was calculated as well as Standard Error of Measurement (SEM) and Minimal Detectable Difference (MDD).

The findings showed varying levels of intra- and inter-session reliability, ranging from poor to excellent. The SEM and MDD values were lower for the intra-session range of motion and grasp evaluation. The reduced reliability can potentially be attributed to skin artifacts, differences in marker placement, and the inherent kinematic variability of finger motion. The proposed marker set shows potential to assess finger postures and analyse activities of daily living, primarily within the context of single session tests.

## 1 Introduction

Quantifying finger kinematics has the potential for enhancing the understanding of finger function, facilitating the design of efficient prosthetics, identifying movement disorders and assessing the impact of rehabilitation interventions [1]. Numerous studies have evaluated finger kinematics using a variety of methods and purposes, including the use of musical instruments [2], during Activities of Daily Living (ADL) [3], and interaction with technological devices [4].

Generally, upper extremity kinematics are more complex when compared to lower leg kinematics [5], therefore, for studies focusing on understanding finger function, reliable characterization of finger biomechanics is required.

Different methods are available for recording finger joint angles in both static and dynamic scenarios, for example wearable gloves [6,7], exoesqueletons [8,9], and motion capture sensors like inertial measurement units [10,11], Xbox Kinect and Leap Motion sensors [[12], [13], [14]].

Motion capture is regarded as the “gold standard” for biomechanical evaluation of human movement. Consequently, this study focuses on the development of a marker set specifically designed for tracking finger motion using motion capture cameras. A review conducted by Reissner et al. [15], examined and compared available marker sets used during ADL, using one, two or three markers (single or in clusters) per segment. The review found no three-marker marker set being used to study finger motion during ADL, and so the potential of such marker sets to gather functional kinematic data remains unknown, despite their potential value in tracking finger movements that occur outside of the standard movement planes. Lee & Jung [16], compared the differences in angles resulting from different marker attachment methods. Their findings suggest that, for dynamic evaluation, the use of three markers per segment is recommended due to reduced skin movement artifacts.

Additionally, Metcalf & Notley [17], recommended the use of marker set concepts with two or more markers, as they exhibit diminished skin movement artifacts, thereby improving measurement accuracy. Two-marker concepts assume that proximal inter-phalangeal (PIP) joint and distal inter-phalangeal (DIP) joint motion occur in a single plane movement of flexion-extension [18], and metacarpophalangeal (MCP) joints have two degrees of freedom (DOFs) [19]. The thumb's interphalangeal (IP) and metacarpophalangeal (MCP) angles have been considered to have one DOF [20,21]. Notably, the interpretation of the carpometacarpal (CMC) joint angle of the thumb can be further elucidated through the utilization of anatomic landmark calibration, which facilitates the analysis of its three DOFs [22].

Considering the advantages offered by the options of using two or three markers per segment, and the lack of experiments undertaken for the study of finger function during ADL using motion capture, we developed a novel hybrid marker set specifically designed for implementation with motion capture cameras.

Currently, there is insufficient information available regarding the reliability of finger kinematic data obtained through motion capture [15,23]. Although there are more studies assessing goniometric reliability [24,25], ensuring reproducibility and meaningful data interpretation requires reliability for assessing reported outcomes [1]. Therefore, the aim of this study was to evaluate the intra- and inter-session reliability of a newly proposed marker set (the Warwick marker set) designed to track finger kinematics using motion capture systems.

The primary goal of this research is to evaluate the reliability of finger kinematic data collected during range of motion movements and selected grasps (for intra-session reliability), repeated over two sessions (for inter-session reliability).

For the segments requiring a three-marker cluster, calibration markers were used for landmark definition. Due to the absence of standardized procedures for placing markers on finger anatomical landmarks, palpation guidelines were developed, and incorporated in this work (see the Supplementary material).

We hypothesize than intra-session reliability will range from moderate to good, while inter-session reliability will be comparatively lower. This expectation stems from the potential drawbacks associated with finger testing and marker set application between sessions. The results of the study allow determination of the capabilities of the proposed marker set in evaluating finger kinematics.

## 2 Methods

The study received ethical approval from the University of Warwick Biomedical & Scientific Research Ethics Committee (BSREC, ID: 77/21–22). Twelve participants (eight male and four females, mean age 21.3 ± 4.2), were recruited for the study and gave informed consent. Each participant attended two data collection sessions at least one day apart to investigate intra- and inter-session reliability. Any participants suffering from any impairments that affect hand and finger motion were excluded from the experiment.

**Fig. 1:**
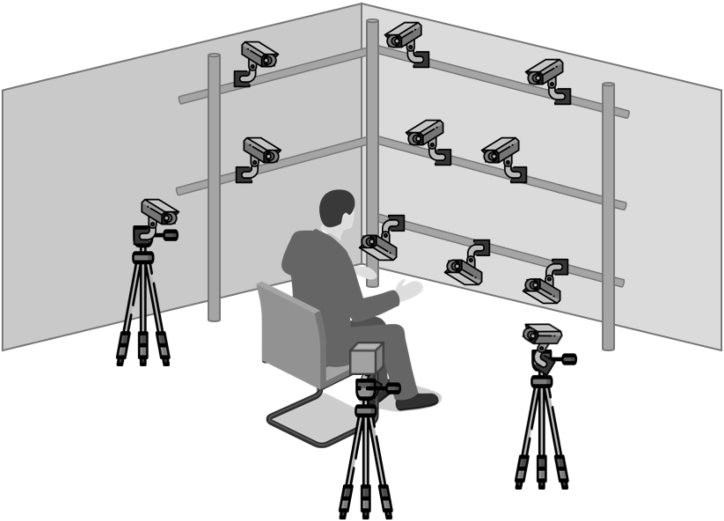
Camera configuration. The cameras were arranged and positioned approximately 90 cm from the intended capture volume.

Data collection took place at the University of Warwick's Gait Laboratory. A motion capture system consisting of 12 MX-T20 cameras (Vicon Motion Systems, LA, USA) collecting at 500Hz was used. The camera setup and markers employed followed the recommendations of Yang et al. [26] for capturing high-resolution movement in small volumes (Fig. 1). Data were captured via reflections from 4 mm spherical markers and two 14 mm markers for the wrist. The marker trajectories were then labelled using Vicon Nexus. Subsequently, the data were saved in Vicon ProCalc, where segment definition and joint angle calculation were performed.

### 2.1 Marker set

**Fig. 2:**
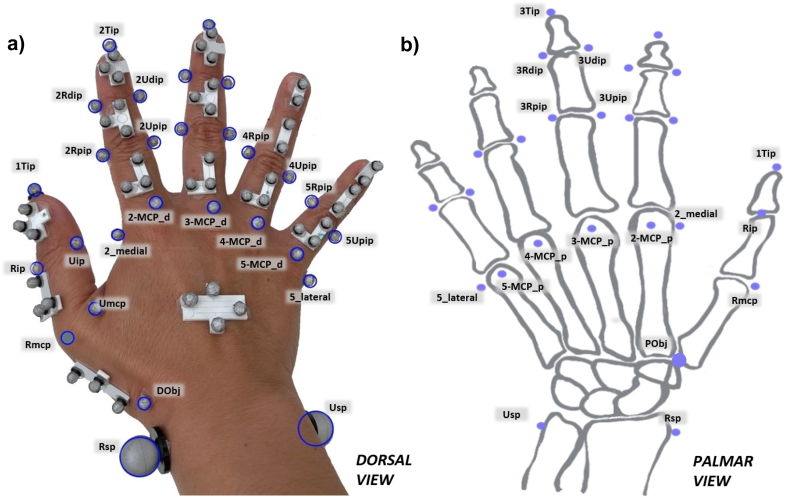
A) Dorsal and b) palmar view of the full marker set with calibration and tracking markers. Blue markers are calibration markers.

A total of 78 markers were positioned on each participant's right hand. A total of 33 calibration markers were positioned on finger joint anatomical landmarks to define rigid segments. Consultant Hand Surgeons from the University Hospitals Coventry and Warwickshire (UHCW), contributed to the development of palpation guidelines (Supplementary material) in order to standardize marker positioning and aid joint identification. Fig. 2a shows the dorsal view of the markers, where blue circles indicate calibration markers. Fig. 2b shows palmar calibration markers.

The calibration markers were used to define the rigid segments of the fingers for kinematic analysis. The segments were defined using non-collinear markers. The anatomical reference planes were defined using a right-handed Cartesian coordinate systems using a Cardan XYZ rotation [27]. The rotation sequence involved initial rotation about the laterally directed axis (X); followed by rotation around the anteriorly directed axis (Y), and finally, rotation around the vertical axis (Z) [28].

The segments defined were.

• The hand,
• For the Index, Middle, Ring, and Little fingers: proximal segments
• For the Index and Middle fingers: Middle and distal segments

**Fig. 3:**
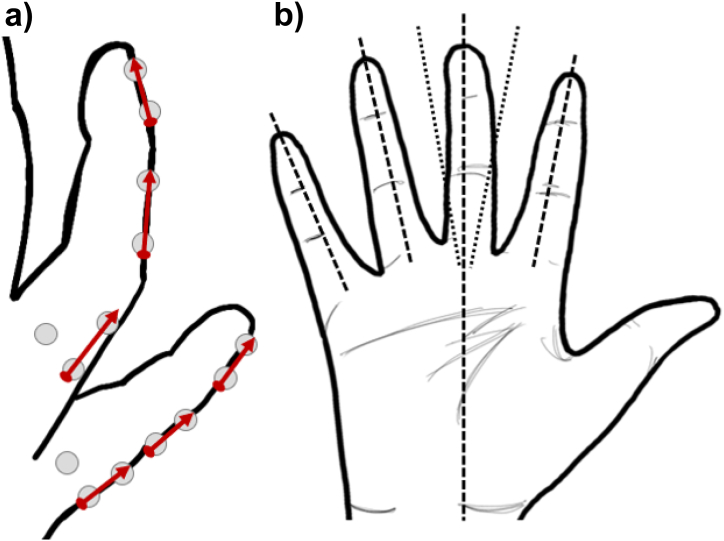
A) Vectors defined for the calculation of flexion/extension of PIP and DIP joints for the ring and little fingers, b) representation of the finger vectors used to calculate abduction/adduction. A reference vector was defined using the midpoint of the Rsp and Usp markers (See Supplementary material) towards the centre of the middle finger PIP joint. For the index, ring, and little fingers, vectors were defined from the MCP joint origin to the PIP joint origin. Angles were calculated using the middle finger static calibration line as a reference.

A total of 45 tracking markers were used. A four-marker cluster was used for the hand segment taking advantage of space for easy marker tracking. Three marker clusters were used for kinematic segments, except for the Middle and distal segments of the Ring and Little fingers where two markers per segment were used. The selection of this approach took into consideration the participants' comfort while wearing the marker set, the limited space available on smaller segments, and consideration of joint angles as having one degree of freedom (flexion-extension). Segment vectors were defined using two collinear markers in the distal, medial, and proximal clusters (Fig. 3a). PIP and DIP joint angles were calculated as the angles between the two vectors.

Abduction/adduction angles were defined as the angle between the fingers’ reference vectors and the Middle finger reference vector position during static calibration (Fig. 3b) respectively. For the thumb carpometacarpal (CMC) joint, rotations around each axis were recorded and are shown as CMC_x, CMC_y and CMC_z. For the rest of the joints, only flexion/extension angles were extracted and reported.

### 2.2 Experimental protocol

**Fig. 4:**
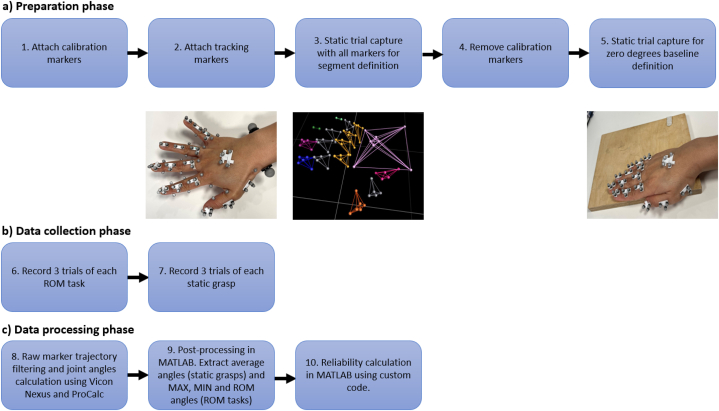
Preparation and static calibration process for data collection.

The preparation of the participants for the data collection took place following the steps outlined in the preparation phase of Fig. 4. First, calibration markers were attached using the palpation guidelines previously mentioned. Second, tracking markers were attached. In step 3, a static trial was recorded with participants wearing all calibration and tracking markers to capture the necessary kinematic segments. After removing calibration markers in step 4, step 5 consisted of establishing a zero-degree baseline for the finger joints during tests by recording a calibration static trial using only tracking markers. Following procedures described by Cook et al. [29], and Nataraj & Li [30], participants utilized a flat, square block of wood, acting as a digit alignment device. In this position, as depicted in Fig. 4a, step 5, participants laid their hand flat with fully adducted fingers, while the thumb remained fully extended and adducted. Finger joint angles were recorded and averaged over 1 s for the normalization of all motion tasks and static grasps.

The data collection phase (Fig. 4b, steps 6 and 7), consisted of recording Range of motion (ROM) tasks and static grasps.

• *Range of motion (ROM) tasks*

**Fig. 5:**
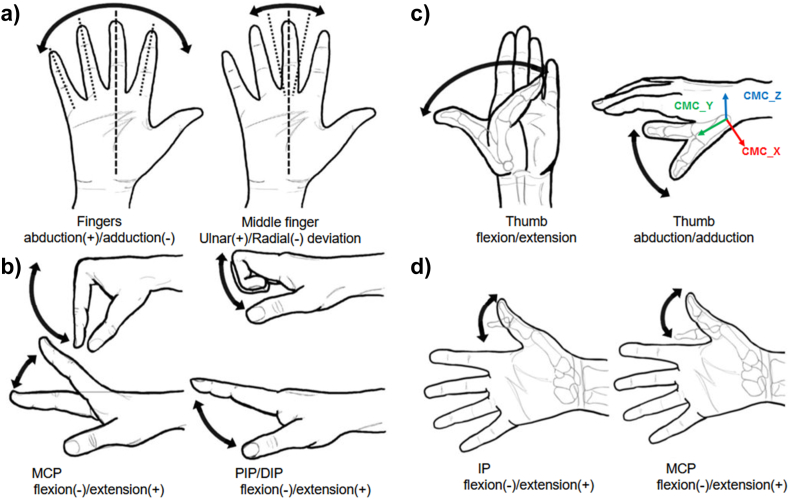
Finger joints motion tasks. Adapted from Hirt et al. [31], Joint rotations signs correspond to the sequence defined by Robertson, 2014, except for thumb flexion/extension and abduction/adduction, which are defined by the CMC joint. a) Index to little finger abduction/adduction was calculated by the radial/ulnar deviation from the line of reference obtained from static calibration. b) MCP, PIP and DIP flexion/extension angles were calculated for index to little fingers. c) Thumb CMC joint kinematics for angle interpretation. d) Thumb's IP and MCP flexion/extension angles.

All participants were asked to perform a series of movements to evaluate joint maximum, minimum and ROM angles. The participants were instructed to flex and extend finger joints as much as possible during these movements. To isolate joint movements, participants performed the movements in different sets, as seen in Fig. 5. Each participant completed three trials for each task.

• *Static grasps*

**Fig. 6:**
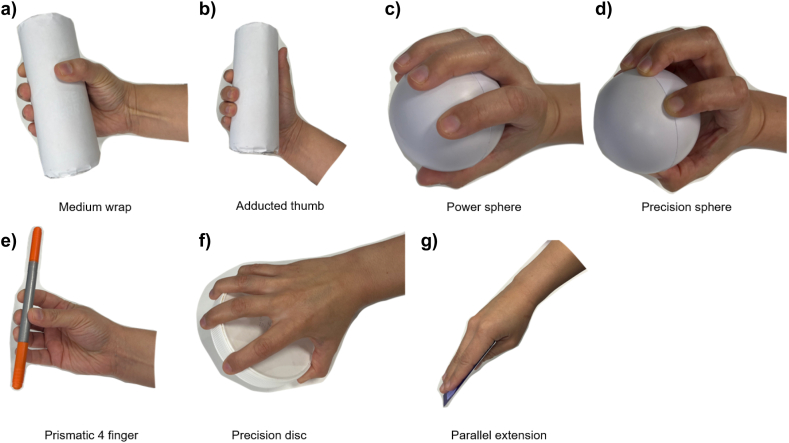
Selected grasps for the static tests. a) Medium wrap involved all fingers wrapping around the cylinder object while b) Adducted thumb required a different thumb position. c) Power sphere grasp involved fingers wrapping the ball whereas d) Precision sphere required only fingertips to hold the ball. For the e) Prismatic 4 finger, f) Precision disc and g) Parallel extension grasps, a marker, a detergent lid and a card were used to accomplish each grasp respectively.

To evaluate full hand kinematic reliability, participants were instructed to hold a series of objects, which remained consistent for all participants and across sessions. The grasps were obtained from the GRASP taxonomy [32] and were chosen based on the following criteria: 1) grasps that allow the recording of all finger segments without overlapping or occlusion; 2) grasps were all fingers are in contact with the object; and 3) commonly observed grasps during household and machining tasks [33]. Participants held the indicated object and maintained the desired grasp for 1 s without moving or changing position (Fig. 6). Three trials per grasp were recorded for each participant.

### 2.3 Data processing and data analysis

As outlined in Fig. 4c, step 8, raw marker trajectories were filtered in Vicon Nexus using a fourth order zero-lag low-pass Butterworth filter with a cut-off 15 Hz frequency. Raw marker trajectory data were filtered to remove any displacement distortion that could result in angle signal peaks during calculation. Finger joint angles were calculated using Vicon ProCalc and then exported to MATLAB R2020b software. Angle data for all trials and tasks were filtered using a zero-lag fourth order low-pass Butterworth filter with a 5Hz cut-off frequency, based on Skogstad et al. [34], recommendations for hand motion tracking (Fig. 4c, step 9). This filtering approach was chosen given its strengths at noise attenuation during hand kinematics motion capture and usefulness at recording free-hand motion. For the range of motion tasks, the maximum, minimum and range of motion angles were extracted. For static grasps, joint angles were averaged over 1 s of recording.

To assess inter-session reliability (Fig. 4c, step 10), a two-way mixed, absolute agreement, average of *K* measurements was used, where *K* = 3 trials per session. For intra-session reliability, three trials from the second session were used using a two-way mixed, absolute agreement, single measures model. The ICC model and type was selected following Koo & Li [34], and Shrout & Fleiss [35], recommendations for assessing test-retest reliability. ICCs were interpreted according to Koo & Li, 2016, where <0.50 represents poor reliability; 0.50–0.74 moderate reliability; 0.75–0.89 good reliability and ≥0.9 excellent reliability.

Standard Error of Measurement (SEM) was calculated using Equation (1), as the square root of the error variance [36] where [37]:

(Equation 1) $$\sqrt{\sigma_{error}^{2}}=\sqrt{\sigma_{bias}^{2}+\sigma_{sample}^{2}}$$

The Minimal Detectable Difference (MDD) was calculated using Equation (2)[38]:

(Equation 2) $$1.96\times \text{SEM}\times \sqrt{2}\;$$

All calculations were performed in MATLAB using custom code written by Kevin Brownhill (Imaging Sciences, KCL, London. kevin.brownhill@kcl.ac.uk) based on Shrout and Fleiss’ original paper [35].

## 3 Results

The intra-session reliability results for the joint angles were consistently higher than the inter-session reliability for both the static and ROM tasks. For the Index finger, ROM task reliability was lower in the inter-session case (ICC = −0.87-0.9) compared to the intra-session case (ICC = 0.72–0.99), with most of ICC values in the good-excellent categories (Table 1). This was also the case for the Middle finger. A similar trend was observed during static grasps, except for the DIP joint, where inter-session reliability was better during power sphere (0.75 > 0.5) and precision sphere grasps (0.76 > 0.64) (Table 1).

**Table 1:** Reliability results for the Index finger.

Range of motion reliability													
		MAX			MIN			ROM					
		ICC	SEM	MDD	ICC	SEM	MDD	ICC	SEM	MDD			
Inter-session	Abduction/adduction	0.50	2.53	7.01	0.28	2.43	6.74	0.39	2.50	6.94			
	MCP flexion/extension	−0.04	6.75	18.70	0.71	4.35	12.05	0.69	6.22	17.23			
	PIP flexion/extension	−0.76	10.19	28.26	0.44	8.14	22.57	−0.22	17.77	49.25			
	DIP flexion/extension	−0.87	12.22	33.86	−0.46	12.27	34.01	0.90	2.88	7.99			
Intra-session	Abduction/adduction	0.92	1.60	4.44	0.72	2.79	7.72	0.78	2.79	7.72			
	MCP flexion/extension	0.88	4.23	11.74	0.93	3.13	8.67	0.91	4.60	12.75			
	PIP flexion/extension	0.99	2.12	5.87	0.91	6.39	17.70	0.97	7.05	19.54			
	DIP flexion/extension	0.99	2.41	6.68	0.96	4.66	12.90	0.83	5.43	15.06			
*Static grasps reliability*													
		DIP Index			PIP Index			MCP Index			ABDADD Index		
		ICC	SEM	MDD	ICC	SEM	MDD	ICC	SEM	MDD	ICC	SEM	MDD
Inter-session	Medium wrap	0.90	1.87	5.18	0.72	2.56	7.11	0.87	3.82	10.59	0.68	3.60	9.98
	Adducted thumb	0.79	2.56	7.11	0.84	2.31	6.40	0.69	5.99	16.59	0.58	4.22	11.69
	Power sphere	0.75	2.53	7.01	0.65	3.09	8.57	0.67	5.50	15.24	0.05	5.61	15.54
	Precision sphere	0.76	1.98	5.47	0.75	3.09	8.57	0.05	5.47	15.17	0.13	4.46	12.37
	Prismatic 4 fingers	0.48	8.95	24.81	0.68	4.19	11.61	0.76	3.81	10.57	0.69	4.55	12.62
	Precision disc	0.73	3.57	9.89	0.75	3.98	11.02	0.66	5.09	14.10	0.51	3.65	10.10
	Parallel extension	0.08	13.57	37.62	0.80	2.47	6.83	0.50	4.21	11.66	0.56	5.05	13.98
Intra-session	Medium wrap	0.91	2.33	6.45	0.82	2.46	6.81	0.94	3.20	8.87	0.82	4.27	11.83
	Adducted thumb	0.87	2.73	7.57	0.74	3.24	8.97	0.91	4.47	12.38	0.79	3.69	10.23
	Power sphere	0.50	5.23	14.49	0.75	3.14	8.71	0.73	7.91	21.93	0.66	6.26	17.36
	Precision sphere	0.64	3.72	10.30	0.78	3.77	10.44	0.65	3.55	9.84	0.94	2.60	7.21
	Prismatic 4 fingers	0.54	19.29	53.47	0.40	9.91	27.47	0.66	6.50	18.01	0.62	9.24	25.62
	Precision disc	0.81	4.24	11.74	0.83	4.07	11.29	0.82	3.98	11.03	0.91	2.38	6.61
	Parallel extension	0.93	1.70	4.70	0.86	2.96	8.21	0.70	5.22	14.48	0.88	4.55	12.62

Table 2 shows a reduced ICC for the parallel extension grasp in the inter-session case of the Middle finger for DIP (−0.17), PIP (0.65) and MCP (0.25) joints when compared to other grasps. Intra-session reliability was generally higher than inter-session reliability except for the power sphere, precision sphere, prismatic 4 fingers and parallel extension grasps. This occurred for the Index (DIP in power and precision sphere, PIP in prismatic 4 fingers, and MCP in prismatic 4 fingers, Table 1), Middle (DIP in precision sphere, PIP in power sphere, precision sphere and prismatic 4 fingers, and MCP in power sphere and precision 4 fingers, Table 2), Ring (all joints, Table 3), and Little (PIP, MCP, and abduction/adduction, Table 4)

**Table 2:** Reliability results for the Middle finger.

Range of motion reliability													
		MAX			MIN			ROM					
		ICC	SEM	MDD	ICC	SEM	MDD	ICC	SEM	MDD			
Inter-session	Radial/Ulnar deviation	0.48	2.55	7.07	0.24	2.64	7.30	−0.05	2.70	7.49			
	MCP flexion/extension	−0.23	6.64	18.41	0.85	3.29	9.13	0.65	6.79	18.81			
	PIP flexion/extension	0.39	3.20	8.88	0.92	2.54	7.04	0.79	4.55	12.62			
	DIP flexion/extension	0.42	2.48	6.87	0.45	5.04	13.96	0.53	5.23	14.49			
Intra-session	Radial/Ulnar deviation	0.88	2.18	6.04	0.77	2.50	6.92	0.74	2.66	7.36			
	MCP flexion/extension	0.88	3.96	10.99	0.92	3.37	9.34	0.90	4.71	13.05			
	PIP flexion/extension	0.79	3.03	8.40	0.82	5.34	14.79	0.80	7.00	19.41			
	DIP flexion/extension	0.76	3.19	8.84	0.77	3.73	10.34	0.72	5.57	15.44			
*Static grasps reliability*													
		DIP Middle			PIP Middle			MCP Middle			RADIAL/ULNAR DEVIATION		
		ICC	SEM	MDD	ICC	SEM	MDD	ICC	SEM	MDD	ICC	SEM	MDD
Inter-session	Medium wrap	0.84	2.39	6.62	0.78	2.44	6.76	0.91	3.22	8.91	0.85	3.86	10.70
	Adducted thumb	0.87	2.22	6.15	0.88	2.15	5.95	0.75	5.02	13.92	0.47	4.93	13.68
	Power sphere	0.88	2.72	7.53	0.52	2.92	8.09	0.78	4.16	11.54	0.44	5.30	14.69
	Precision sphere	0.68	2.45	6.78	0.87	2.63	7.30	0.61	3.76	10.43	0.27	4.14	11.48
	Prismatic 4 fingers	0.80	2.99	8.28	0.87	3.34	9.27	0.46	4.64	12.87	0.62	4.79	13.28
	Precision disc	0.74	2.78	7.70	0.87	2.44	6.77	0.82	3.74	10.37	0.29	3.40	9.43
	Parallel extension	−0.17	8.12	22.50	0.65	3.16	8.76	0.25	4.23	11.73	0.52	5.39	14.95
Intra-session	Medium wrap	0.93	2.00	5.53	0.95	1.41	3.92	0.95	3.03	8.40	0.96	2.40	6.64
	Adducted thumb	0.87	2.73	7.56	0.84	3.00	8.33	0.92	3.82	10.60	0.85	3.27	9.07
	Power sphere	0.84	3.39	9.40	0.15	4.98	13.81	0.76	6.34	17.57	0.74	4.71	13.04
	Precision sphere	0.23	7.18	19.90	0.71	4.78	13.24	0.63	3.55	9.83	0.94	1.80	4.98
	Prismatic 4 fingers	0.86	3.24	8.99	0.38	12.81	35.51	0.43	6.81	18.88	0.30	9.53	26.42
	Precision disc	0.59	4.72	13.09	0.74	4.03	11.16	0.88	3.54	9.81	0.88	2.21	6.13
	Parallel extension	0.88	1.06	2.93	0.83	2.52	6.97	0.67	5.50	15.24	0.89	4.70	13.03

**Table 3:** Reliability results for the Ring finger.

Range of motion reliability													
		MAX			MIN			ROM					
		ICC	SEM	MDD	ICC	SEM	MDD	ICC	SEM	MDD			
Inter-session	Abduction/adduction	0.69	2.55	7.07	0.40	3.25	9.00	0.68	1.62	4.50			
	MCP flexion/extension	−0.46	5.90	16.35	0.34	5.36	14.86	0.26	7.34	20.34			
	PIP flexion/extension	0.37	3.31	9.17	0.83	4.02	11.14	0.64	4.76	13.19			
	DIP flexion/extension	−0.36	2.16	5.99	0.85	3.74	10.36	0.77	4.51	12.50			
Intra-session	Abduction/adduction	0.91	1.89	5.23	0.93	1.97	5.45	0.61	2.96	8.21			
	MCP flexion/extension	0.88	3.39	9.39	0.87	4.24	11.76	0.84	5.58	15.47			
	PIP flexion/extension	0.97	1.46	4.05	0.87	5.68	15.75	0.80	6.16	17.08			
	DIP flexion/extension	0.80	1.38	3.81	0.94	3.37	9.34	0.95	3.21	8.89			
*Static grasps reliability*													
		DIP Ring			PIP Ring			MCP Ring			ABDADD Ring		
		ICC	SEM	MDD	ICC	SEM	MDD	ICC	SEM	MDD	ICC	SEM	MDD
Inter-session	Medium wrap	0.63	4.68	12.97	0.31	3.84	10.64	0.73	5.12	14.20	0.58	5.60	15.52
	Adducted thumb	0.62	5.16	14.32	0.50	3.90	10.82	0.53	4.25	11.78	0.32	5.08	14.09
	Power sphere	0.61	5.04	13.96	0.85	2.55	7.06	0.83	4.17	11.56	0.19	5.49	15.21
	Precision sphere	0.70	4.29	11.88	0.81	3.69	10.23	0.34	7.00	19.40	0.60	3.84	10.64
	Prismatic 4 fingers	0.86	3.68	10.20	0.69	5.88	16.29	0.72	9.08	25.18	0.57	6.00	16.64
	Precision disc	0.67	4.74	13.14	0.68	3.44	9.54	0.81	4.09	11.35	0.60	3.50	9.70
	Parallel extension	0.86	1.15	3.20	0.76	2.98	8.26	0.78	6.35	17.60	0.69	6.44	17.84
Intra-session	Medium wrap	0.98	1.79	4.97	0.97	1.09	3.02	0.96	2.83	7.85	0.94	3.15	8.74
	Adducted thumb	0.94	3.15	8.74	0.87	3.21	8.91	0.86	3.82	10.58	0.90	2.76	7.64
	Power sphere	0.96	2.32	6.43	0.64	6.55	18.15	0.80	6.99	19.38	0.66	6.23	17.26
	Precision sphere	0.84	4.51	12.50	0.86	4.59	12.73	−0.07	27.81	77.09	0.77	4.67	12.94
	Prismatic 4 fingers	0.66	8.50	23.56	0.86	5.66	15.68	0.45	20.39	56.52	0.68	7.30	20.22
	Precision disc	0.91	4.15	11.51	0.90	3.36	9.32	0.87	4.15	11.51	0.97	1.20	3.33
	Parallel extension	0.89	1.23	3.42	0.42	7.81	21.65	0.07	22.40	62.08	0.47	13.41	37.18

**Table 4:** Reliability results for the Little finger.

Range of motion reliability													
		MAX			MIN			ROM					
		ICC	SEM	MDD	ICC	SEM	MDD	ICC	SEM	MDD			
Inter-session	Abduction/adduction	0.74	3.47	9.62	0.39	3.36	9.31	0.90	1.88	5.22			
	MCP flexion/extension	0.58	4.73	13.12	−0.02	6.90	19.12	0.63	7.14	19.80			
	PIP flexion/extension	0.62	3.28	9.09	0.83	4.11	11.39	0.81	5.34	14.82			
	DIP flexion/extension	0.74	2.39	6.64	0.83	4.63	12.82	0.79	4.53	12.54			
Intra-session	Abduction/adduction	0.97	1.72	4.76	0.95	1.69	4.69	0.92	2.29	6.34			
	MCP flexion/extension	0.91	3.57	9.91	0.88	4.92	13.63	0.88	5.70	15.80			
	PIP flexion/extension	0.91	2.10	5.81	0.78	6.87	19.04	0.82	7.67	21.25			
	DIP flexion/extension	0.96	1.25	3.46	0.95	3.77	10.45	0.93	3.89	10.79			
*Static grasps reliability*													
		DIP Little			PIP Little			MCP Little			ABDADD Little		
		ICC	SEM	MDD	ICC	SEM	MDD	ICC	SEM	MDD	ICC	SEM	MDD
Inter-session	Medium wrap	0.64	4.70	13.03	0.29	4.13	11.45	0.80	5.37	14.89	0.66	5.85	16.21
	Adducted thumb	0.87	3.83	10.61	0.60	4.15	11.49	0.73	3.62	10.04	0.50	4.76	13.19
	Power sphere	0.86	4.11	11.39	0.42	4.39	12.17	0.74	5.18	14.37	0.69	5.44	15.09
	Precision sphere	0.82	2.88	7.97	0.77	2.34	6.50	0.30	10.27	28.46	0.86	3.57	9.90
	Prismatic 4 fingers	0.91	2.60	7.19	0.91	2.67	7.39	0.27	10.05	27.85	0.61	6.49	18.00
	Precision disc	0.75	4.27	11.84	−0.07	5.06	14.02	0.49	5.60	15.52	0.61	5.42	15.01
	Parallel extension	0.78	1.46	4.04	0.49	4.07	11.29	0.79	10.99	30.50	0.54	9.52	26.38
Intra-session	Medium wrap	0.95	2.29	6.34	0.94	2.48	6.86	0.92	5.15	14.28	0.97	2.81	7.78
	Adducted thumb	0.87	4.91	13.61	0.76	4.74	13.13	0.84	4.49	12.45	0.90	2.65	7.34
	Power sphere	0.96	2.49	6.89	0.34	10.43	28.90	0.61	13.12	36.36	0.83	6.02	16.69
	Precision sphere	0.94	2.34	6.48	0.43	7.00	19.40	0.35	24.24	67.20	0.78	6.64	18.41
	Prismatic 4 fingers	0.96	2.04	5.66	0.79	6.33	17.55	0.56	14.58	40.43	0.63	9.52	26.37
	Precision disc	0.89	4.08	11.31	0.75	4.80	13.30	0.83	5.17	14.34	0.90	3.86	10.69
	Parallel extension	0.95	0.87	2.40	0.05	9.43	26.15	0.20	37.74	104.60	0.58	12.98	35.98

### 3.1 ROM tasks

• *Inter-session:*

Finger abduction-adduction Inter-Class Correlation (ICC) values ranged from poor to excellent for all fingers, with notably low values for the Index, Middle and Ring fingers (Table 1, Table 2, Table 3).

The reliability of MCP joint angles during flexion-extension was generally lower for the maximum angle compared to the minimum angle (Table 1, Table 2, Table 3, Table 4), except for the Little finger MCP joint (Table 4).

It was noted that when the reliability of the maximum or minimum angle was low, it also affected the corresponding reliability of the ROM values.

Conversely, the reliability of flexion-extension in DIP joints was primarily poor for the maximum and minimum angles, although the ROM reliability reached moderate to good values, except for the Little finger DIP joint (Table 4), where all reliability values ranged between moderate and good. As for the PIP joints, ROM and minimum angle reliability were higher (ICC = 0.64–0.92) for all fingers except for the Index PIP joint (Table 1).

Regarding thumb abduction-adduction, maximum CMC angles’ reliability was moderate to excellent (ICC = 0.67–0.97) (Table 5), but poor for the minimum and ROM reliability. The reliability of CMC angles during flexion-extension varied from poor to excellent. The MCP flexion-extension reliability was excellent for the maximum angle and poor for the minimum angle and ROM. IP flexion-extension, the reliability was good for ROM but poor for both maximum and minimum angle reliability.

**Table 5:** Reliability results for the Thumb.

Range of motion reliability																	
			MAX			MIN			ROM								
			ICC	SEM	MDD	ICC	SEM	MDD	ICC	SEM	MDD						
Inter-session	Abduction/adduction	CMC_x	0.74	35.83	99.31	−2.34	48.43	134.25	−0.36	70.86	196.42						
		CMC_y	0.67	15.34	42.52	−0.42	26.24	72.75	−0.43	16.04	44.47						
		CMC_z	0.97	9.33	25.85	0.13	45.53	126.21	−0.26	33.97	94.16						
	Flexion/extension	CMC_x	0.81	11.29	31.29	0.97	11.2	31.04	0.97	10.31	28.58						
		CMC_y	0.81	12.17	33.72	0.73	17.66	48.96	0.41	10.93	30.29						
		CMC_z	0.31	57.82	160.27	0.74	40.55	112.41	0.18	28.40	78.72						
	MCP flexion/extension		0.96	6.64	18.42	−0.18	7.98	22.12	0.17	12.40	34.36						
	IP flexion/extension		−0.17	10.56	29.28	0.51	25.59	70.94	0.88	7.29	20.21						
Intra-session	Abduction/adduction	CMC_x	0.99	2.67	7.41	0.99	1.94	5.38	0.99	2.50	6.92						
		CMC_y	0.99	1.72	4.77	0.98	3.03	8.40	0.97	3.46	9.59						
		CMC_z	0.99	2.21	6.12	0.99	2.17	6.00	0.99	2.92	8.10						
	Flexion/extension	CMC_x	0.97	6.57	18.21	0.99	3.65	10.10	0.99	8.21	22.76						
		CMC_y	0.99	2.09	5.80	0.99	3.38	9.36	0.97	3.85	10.68						
		CMC_z	0.99	1.80	5.00	0.99	2.04	5.65	0.92	3.02	8.38						
	MCP flexion/extension		0.65	23.91	66.28	0.94	3.84	10.64	0.92	22.98	63.69						
	IP flexion/extension		0.88	7.11	19.70	0.99	3.48	9.65	0.93	7.53	20.86						
*Static grasps reliability*																	
			MCP Thumb			IP			CMC_x			CMC_y			CMC_z		
			ICC	SEM	MDD	ICC	SEM	MDD	ICC	SEM	MDD	ICC	SEM	MDD	ICC	SEM	MDD
Inter-session	Medium wrap		−0.07	81.95	227.15	0.65	20.22	56.03	0.27	34.02	94.29	0.39	9.40	26.05	0.12	27.34	75.78
	Adducted thumb		0.03	86.30	239.20	−0.98	23.88	66.19	−0.04	63.05	174.77	0.54	9.43	26.13	0.42	30.30	83.99
	Power sphere		0.20	60.14	166.70	0.64	19.85	55.02	0.01	52.31	145.00	0.43	10.19	28.24	0.47	26.39	73.14
	Precision sphere		0.04	86.54	239.89	0.65	20.42	56.59	0.45	34.63	96.00	0.44	10.35	28.68	0.35	27.21	75.43
	Prismatic 4 fingers		0.22	39.42	109.27	0.35	20.79	57.62	−0.43	31.40	87.04	0.41	11.11	30.79	0.17	26.78	74.23
	Precision disc		0.20	75.39	208.97	0.59	19.25	53.35	−0.22	51.45	142.62	0.29	11.97	33.18	0.48	25.53	70.77
	Parallel extension		0.27	35.51	98.42	−0.54	23.43	64.94	0.56	39.76	110.22	0.44	11.66	32.31	0.42	27.23	75.49
Intra-session	Medium wrap		0.97	2.26	6.27	0.96	4.44	12.30	0.97	14.70	40.74	0.32	8.79	24.36	0.99	3.91	10.83
	Adducted thumb		0.17	16.07	44.54	0.90	5.49	15.22	0.93	3.22	8.92	0.91	3.27	9.07	0.99	3.48	9.64
	Power sphere		0.99	6.77	18.78	0.89	7.39	20.48	0.99	5.02	13.92	0.81	3.48	9.64	0.99	3.62	10.02
	Precision sphere		0.99	2.49	6.90	0.86	9.45	26.19	0.99	3.25	9.00	0.96	1.81	5.02	0.99	2.52	6.97
	Prismatic 4 fingers		0.97	3.29	9.13	0.90	9.21	25.54	0.99	2.78	7.71	0.93	2.24	6.22	0.99	2.81	7.79
	Precision disc		0.99	4.31	11.96	0.82	8.15	22.58	0.82	7.92	21.94	0.96	2.23	6.19	0.99	6.14	17.01
	Parallel extension		0.99	3.32	9.21	0.87	6.96	19.28	0.95	6.77	18.77	0.95	2.57	7.12	0.98	7.37	20.44

All the MDD and SEM values for thumb kinematics were higher (SEM = 6.6–70.9, MDD = 18.4–196.4) compared to the Index, Middle, Ring, and Little fingers (SEM = 1.6–12.2, MDD = 4.5–49.2).

• *Intra-session:*

The reliability of kinematics for all fingers, including all joints and angles, ranged from moderate to excellent. Consistent with the inter-session findings, the SEM and MDD values for the Index, Middle, Ring, and Little fingers were lower (SEM = 1.25–7.67, MDD = 3.46–21.25) (Table 1, Table 2, Table 3, Table 4) compared to the thumb kinematics (SEM = 1.72–23.91, MDD = 4.77–66.28) (Table 5). Despite improved kinematic reliability results for the thumb in the intra-session data compared to the inter-session data, the SEM and MDD values remained high, despite moderate to excellent ICC values.

### 3.2 Static grasps

• *Inter-session:*

The Index finger kinematic reliability during static grasps was poor to good (ICC = 0.05–0.9) (Table 1). The DIP joint displayed the lowest reliability and larger SEM and MDD values, mainly for the prismatic 4 fingers and parallel extension grasps. Conversely, the Middle finger had poor to excellent reliability (ICC = −0.17-0.91) (Table 2). The MDD values were higher for the MCP joint and abduction/adduction. On the other hand, the largest MDD value for the Middle finger was for the DIP joint during the parallel extension grasp. Regarding the Ring finger, reliability results were poor to good (ICC = 0.19–0.86) (Table 3). The MDD values were higher for the PIP and MCP joints for the prismatic 4 fingers grasp and for the MCP and adduction/adduction during parallel extension grasps. The reliability results for the Little finger varied from poor to excellent (ICC = −0.07-0.91) (Table 4). Reliability was lower for the MCP joint, with higher MDD values for the precision sphere, prismatic 4 fingers and parallel extension grasps, particularly during parallel extension in cases of abduction/adduction.

In comparison to the other fingers, the thumb kinematic reliability was lower during static grasps, ranging from poor-moderate (ICC = −0.98-0.65), and displayed higher SEM (9.4–86.54) and MDD (26.05–239.89) values (Table 5).

• *Intra-session:*

Index finger reliability results ranged from poor to excellent (ICC = 0.4–0.94) (Table 1). In terms of MDD values, the prismatic four-finger grasp exhibited higher values across all joints. Similarly, the Middle finger yielded poor to excellent reliability results (ICC = 0.15–0.96) with higher MDD values observed for the prismatic four fingers grasp in the PIP, MCP and abduction/adduction, as well as for the precision sphere in the DIP joint (Table 2). Ring finger reliability results ranged from poor to excellent (ICC = −0.07-0.98) (Table 3), displaying larger SEM and MDD values for the MCP joint during the precision sphere, prismatic four fingers and parallel extension grasps. Likewise, the Little finger exhibited poor to excellent reliability (ICC = 0.05–0.96) (Table 4), with higher SEM and MDD values for the MCP joint during the power sphere, precision sphere, prismatic 4 fingers and parallel extension grasps.

As for the thumb, its reliability results ranged from poor to excellent (ICC = 0.17–0.99) (Table 5). The largest SEM and MDD values were observed for the medium wrap CMC_x and CMC_y values and for CMC_x during parallel extension and precision disc grasps. The largest SEM (16.1) and MDD (44.5) values were for the MCP joint during the adducted thumb grasp.

## 4 Discussion

A marker set was developed for evaluating finger biomechanical function using motion capture systems. This study aimed to establish a comprehensive marker set for the hand and assess its measurement accuracy and reliability. Results obtained partially confirm the experimental hypothesis, indicating that inter-session reliability was lower compared to intra-session reliability. However, both intra and inter-session reliability results across the studies varied from poor to excellent.

Joint ROM measurement is important for clinicians as it serves as an assessment metric, which provides insights into the effects of an intervention [39]. Index, Middle, Ring, and Little fingers’ ROM SEM results ranged between 1.62° and 17.77° for the inter-session case and 2.29–7.67° for intra-session case. SEM values larger than 5° were observed for the Index PIP, Middle MCP, Ring MCD, Little MCP and Little PIP (inter-session) and for the Index DIP, PIP, Middle DIP, PIP, Ring MCP, PIP, Little MCP and PIP (intra-session) joints. Previous marker set evaluations of reliability, akin to manual goniometry, have considered a 5° accuracy threshold [40]. Intra-session values exceeded the 5° threshold by no more than 2.7°, indicating a higher accuracy in ROM measurements. However, it is noteworthy that for the thumb, the ROM and SEM reached up to 70.86° for inter-session and 23.91° for intra-session data, suggesting that ROM measurements for this finger are less accurate using the present method.

The reliability during static grasps appears to depend on the finger posture required by each grasp. Distal joints exhibited larger SEM and MDD values, particularly for the prismatic 4 fingers and parallel extension grasps in the inter-session case, and for MCP and PIP joints during the precision sphere, power sphere, prismatic 4 fingers and parallel extension grasps for the intra-session case. The lower repeatability of prismatic four-finger and precision sphere grasps may be attributed to variations in fingertip positioning while holding the object, resulting in trial-to-trial variability. Furthermore, lower reliability was observed for the thumb's MCP joint during the adducted thumb grasp and for all fingers during the parallel extension grasp, as these grasping configurations are susceptible to the gimbal lock effect, where finger joints approach an extended position close to 0°.

When averaged across all joints, the SEM and MDD values for the intra-session data were 4.27° and 11.83°, respectively, compared to 11.23° and 31.13° for the inter-session data. These intra-session results align with a previous study [15], where the marker set demonstrated SEM ranging from 2.1° to 5° and MDD ranging from 5° to 16° for test-retest results. It is important to note that alternative marker sets are more robust for between-day examinations, allowing for better recognition of smaller changes in mobility within a day. However, only the aforementioned marker set has evaluated reliability and accuracy during kinematic tasks using motion capture systems. Therefore, caution should be exercised when interpreting changes over different sessions as true change or as measurement error when employing this method in a clinical setting.

Several factors can limit the reliability of the studied measures. Finger joint active angle measurement, as indicated by previous research [41], is a highly complex process that presents lower reproducibility compared to simpler joints [42]. This is attributed to the involvement of multiple muscles crossing the joints and tendon gliding [43]. Unlike other joints or structures that provide physical limitations, finger joint movement is relatively unrestricted, making it less reliable compared to the range of motion (ROM) measurements of simple hinge joints [24,44].

The primary objective of the present study was to establish a standardized procedure for capturing finger kinematic data and evaluating maximum, minimum, and ROM angles. However, it should be noted that humans rarely perform movements at their maximum or minimum amplitude, and such tasks are often poorly controlled [45]. Instead, we recommend further evaluation of marker sets in representative movements derived from ADL with defined stages, for instance, systematically recording the reach, grasp, and release phases during an activity like pouring water into a cup would provide valuable insights for future investigations.

The lower inter-session reliability observed in our study may be reminiscent of the lower between-rater reliability commonly observed during goniometric measurements [25]. Therefore, we recommend primarily implementing the methods described in this paper on a single-session basis to enhance reliability and minimize potential sources of error.

Further investigation is warranted to explore the rotation sequence of thumb angles, specifically to identify positions that may trigger the gimbal lock effect and develop thumb-specific rotation sequences to mitigate its occurrence [22,[46], [47], [48]]. It is not recommended to employ reduced marker sets for the thumb as they overlook its anatomical considerations the three-dimensionality nature of its movements.

The limitations of this study include its modest number of participants and the reduced dynamic evaluation of finger function. Further studies could involve the examination of a broader range of hand motions relevant to ADL. Another limitation lies in the use of calibration markers to define segments can introduce errors due to variations in marker placement across sessions [49]. Conversely, the application of surface markers based on palpation introduces some level of inaccuracy in determining the precise location of underlying bone structures [50]. Mixed methods combining imaging and palpation techniques can be useful in mitigating marker placement error, and it is particularly suitable for clinical settings equipped with readily accessible 3D imaging equipment [51]. By employing such mixed methods, researchers and clinicians can improve accuracy and reduce uncertainties associated with marker placement.

On the other hand, this study's strengths lie in its innovative evaluation of both static and dynamic finger kinematics using a novel marker set concept.

In conclusion, intra-session ICC results indicate that a mixed marker set concept is sufficiently reliable when assessing finger joint angles in single-session experiments. The hypothesis was confirmed with inter-session reliability being lower than intra-session reliability. We suggest that the application of The Warwick marker set aligns with the research question at hand, preferably in the context of single-session evaluations.

## Data availability statement

Data will be available upon reasonable request. Currently the same dataset is being used for another analysis that will be published, therefore data will not be available for sharing until after this study is published.

## CRediT authorship contribution statement

**Manuela Paulina Trejo Ramirez:** Writing – original draft, Software, Methodology, Investigation, Formal analysis, Data curation, Conceptualization. **Neil Evans:** Writing – review & editing, Supervision, Conceptualization. **Matthew Venus:** Writing – review & editing, Writing – original draft, Supervision, Conceptualization. **Joseph Hardwicke:** Writing – review & editing, Conceptualization. **Michael Chappell:** Writing – review & editing, Writing – original draft, Supervision, Project administration, Methodology, Funding acquisition, Conceptualization.

## Declaration of competing interest

The authors declare that they have no known potential competing financial interests or personal relationships with other people and organizations that could inappropriately influence the work reported in this paper.
