# Supplementary material for: Reliability, accuracy, and minimal detectable difference of a mixed concept marker set for finger kinematic evaluation
Source: Heliyon. 2023 Oct 31;9(11):e21608. doi: 10.1016/j.heliyon.2023.e21608 (PMC10658241; doi:10.1016/j.heliyon.2023.e21608)

**Supplementary material**

**Palpation notes**

| Segment | Calibration markers | | Tracking markers |
| --- | --- | --- | --- |
| Hand | **2_Medial** | Radial aspect second metacarpal head | 3-4 marker cluster on the dorsal aspect of the hand |
|  | **5_Lateral** | Ulnar aspect fifth metacarpal head |  |
|  | **Rsp** | Radial styloid process |  |
|  | **Usp** | Ulnar styloid process |  |
| Fingers | **#-MCP_d** | Metacarpal phalangeal joint, dorsal | Thumb, Index, and middle fingers:  3 marker cluster on the proximal, middle, and distal phalanx  Ring and little fingers:  3 marker cluster on the proximal phalanx  2 marker cluster on middle and distal phalanx |
|  | **#-MCP_p** | Metacarpal phalangeal joint, palmar |  |
|  | **#Rpip, #Upip** | Radial and ulnar aspects of the PIP joint |  |
|  | **#Rdip, #Udip** | Radial and ulnar aspects of the DIP joint |  |
|  | **#Tip** | Fingertip |  |
| Thumb | **Dobj**  **Pobj** | Carpometacarpal joint  Dorsal aspect  Palmar aspect | 3 marker cluster on the first metacarpal  3 marker cluster on proximal phalanx  3 marker cluster on distal phalanx |
|  | **Rmcp**  **Umcp** | Metacarpal joint markers  Radial aspect  Ulnar aspect |  |
|  | **Rip/ Uip** | Radial/ulnar aspect of the IP joint |  |
|  | **1Tip** | Thumb fingertip |  |

**Hand segment**

**Calibration markers**

- **2_medial**: *When a loose fist is made, the point is on the radial side of the distal end of the second metacarpal bone.*
- Hand in relaxed position. Moving laterally from the “knuckle”, find the most prominent aspect of the metacarpal head.


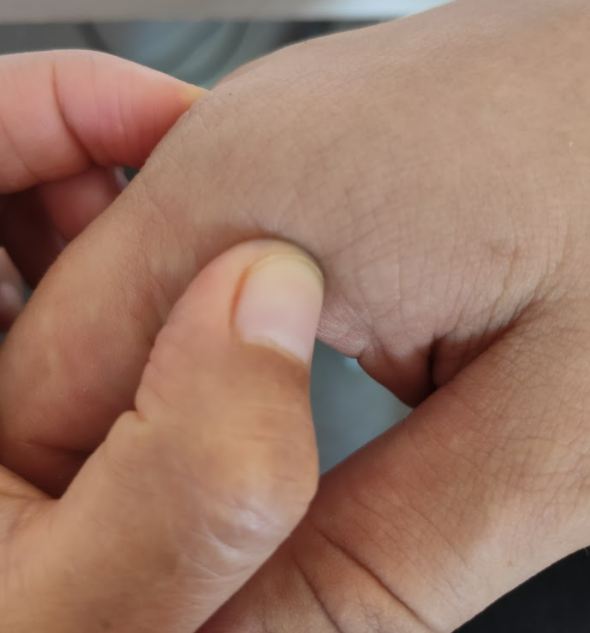

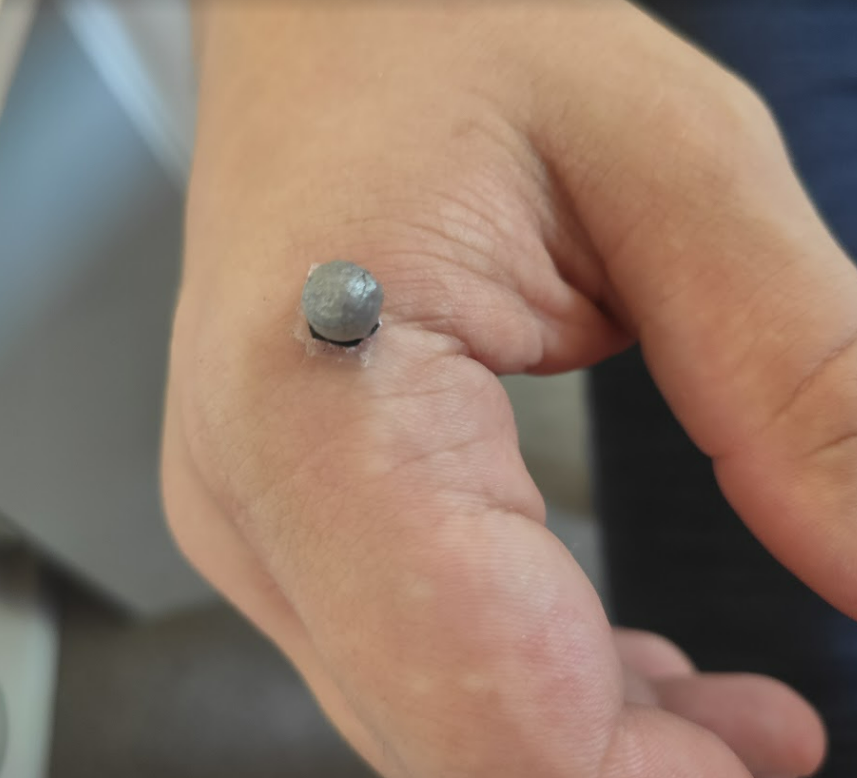


- **5_lateral**: Hand in relaxed position. Moving laterally from the “knuckle”, find the most prominent aspect of the metacarpal head.
- For both metacarpal heads, the marker goes before the “hollow” space between the head and the base of the proximal phalanx.


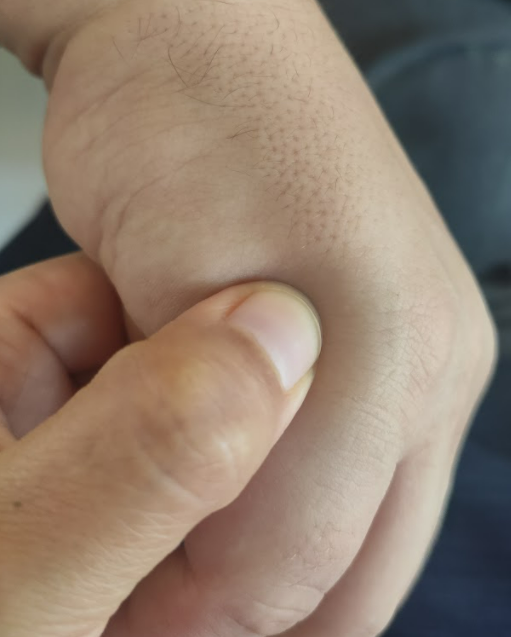

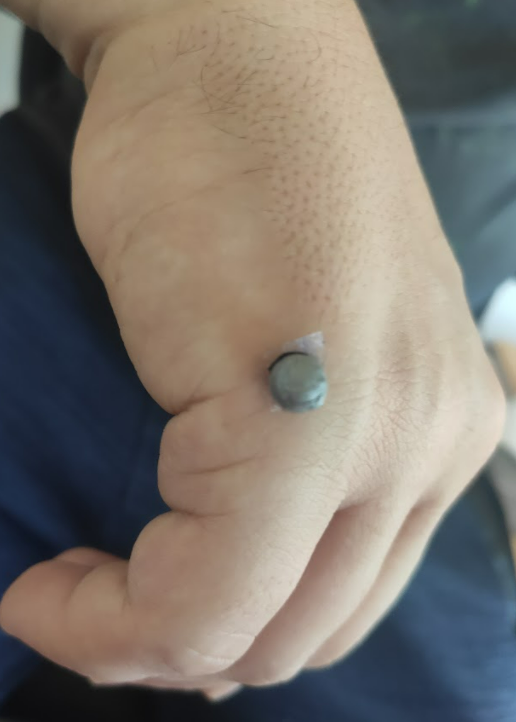


- **Rsp:** *When the thumb is extended, the anatomic snuffbox is easy to see, as it forms a hollow space. The large, blunt radial styloid process can be felt as a clear bony edge*.


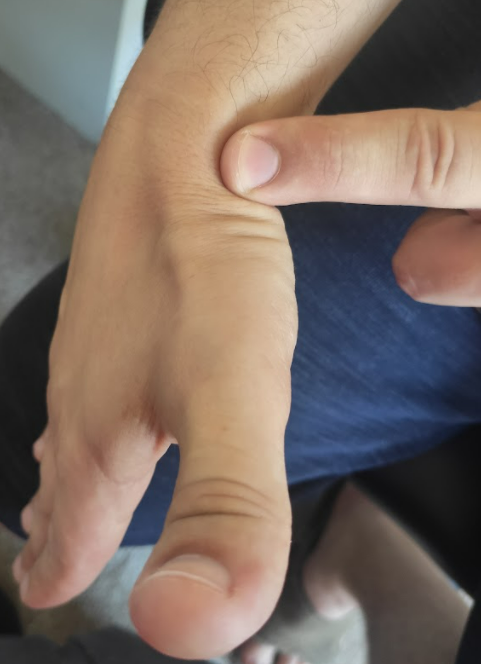

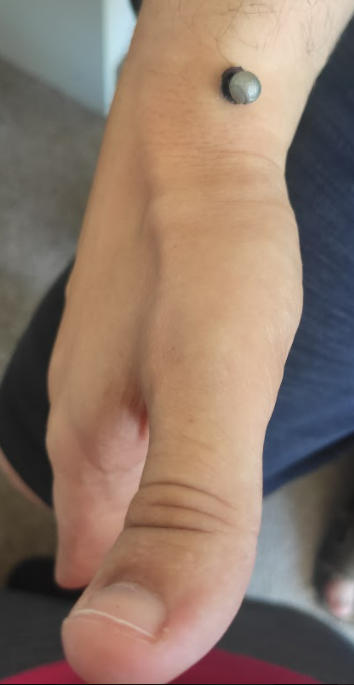


- **Usp:** *At the ulnar head, the ulnar styloid process can be easily palpated as a rod-shaped process.* Bone limit before a “hollow” in between the Ulna and the Triquetrum


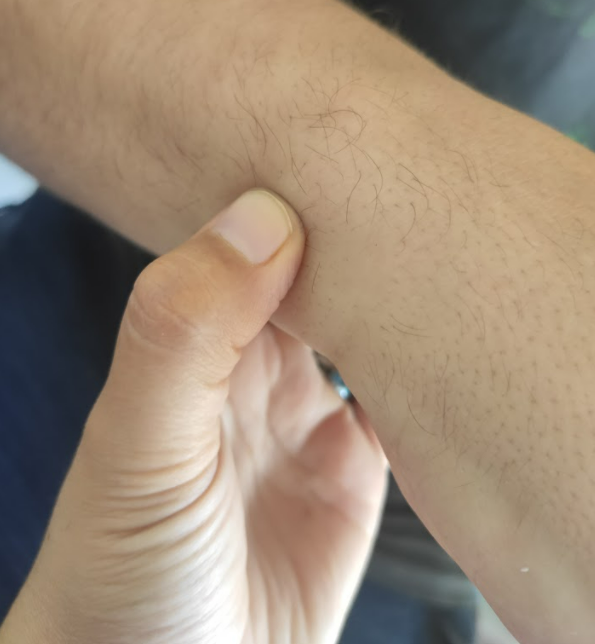

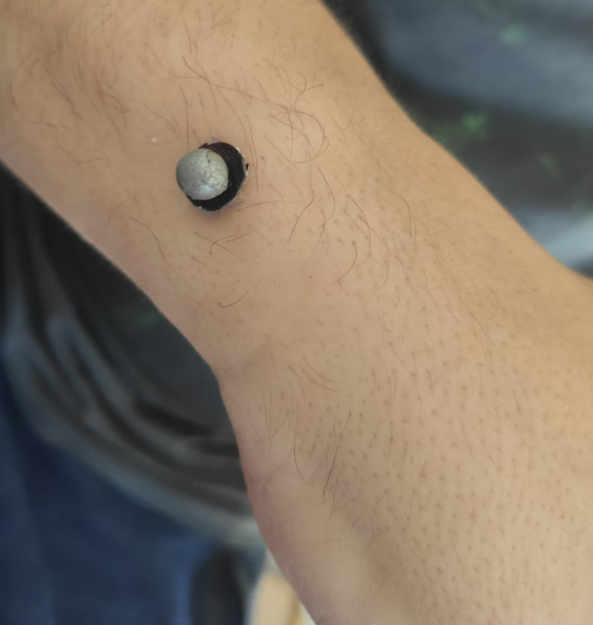


**Tracking markers**

- 3-4 marker cluster plate on the dorsal aspect of the palm. Located on an area with low skin movement.


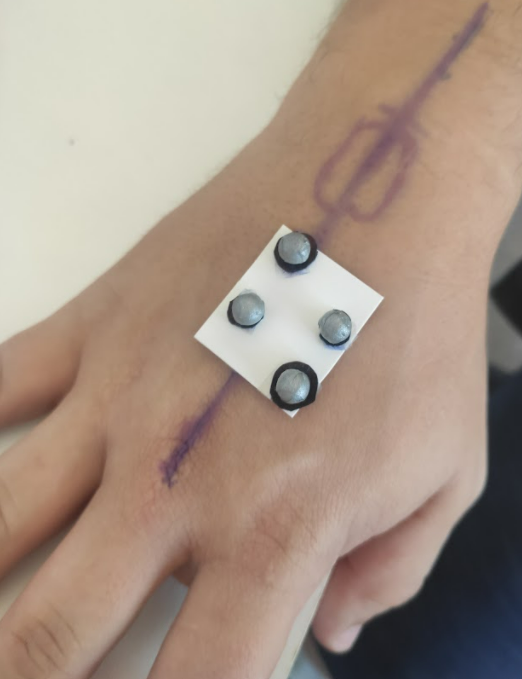


**Finger segments**

**Calibration markers**

- **#-MCP_d**: Below the apex of the metacarpal head, going from proximal to distal. The joint can be palpated as a hollow space, dorsal view. *Site of insertion of extensor tendons*
- **#-MCP_p:** palmar side of the metacarpal head. Using the palmar distal crease as a reference. When flexing the finger, *palpate the site of insertion of flexor tendon.*


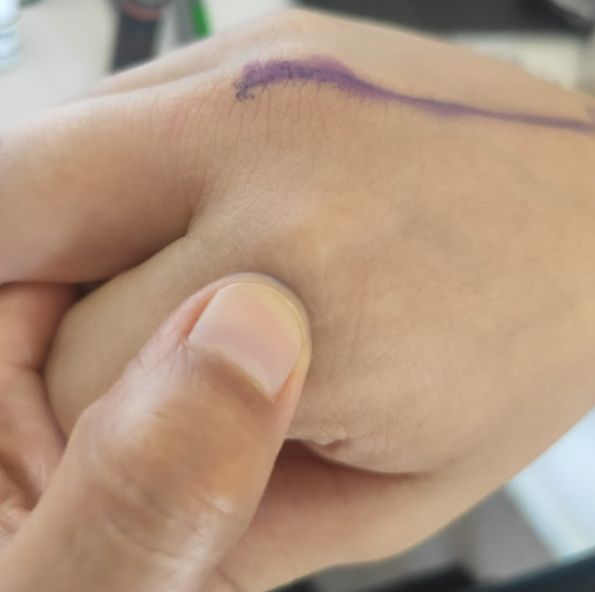

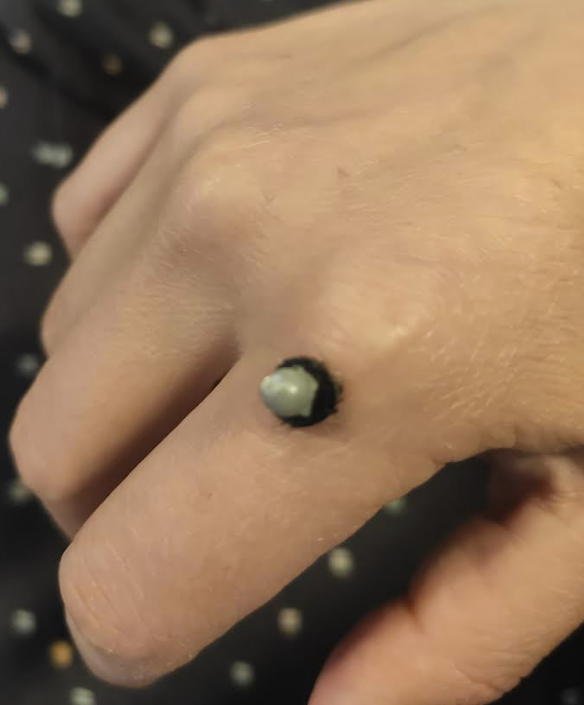


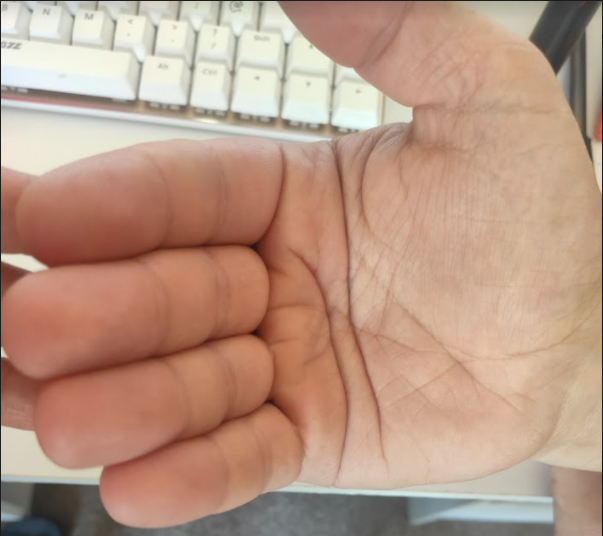

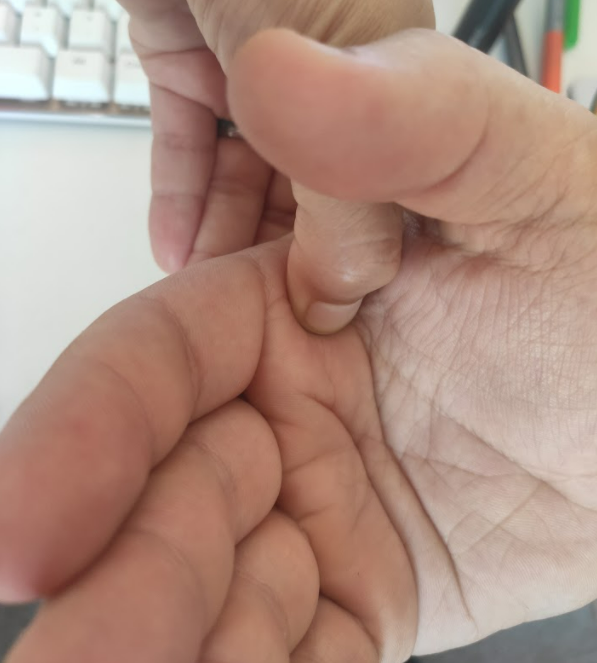

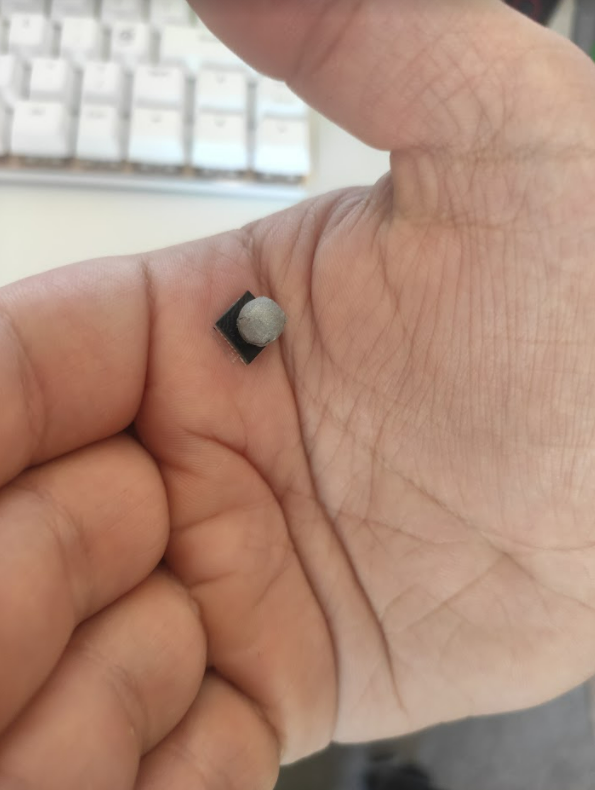


- **#Rpip, #Upip, #Rdip, #Udip**: Markers are positioned on a virtual dot on top of the crease when the finger is fully flexed. Markers can be adjusted by palpating the side of the phalanx bone and position them on the most prominent aspect of the bone.


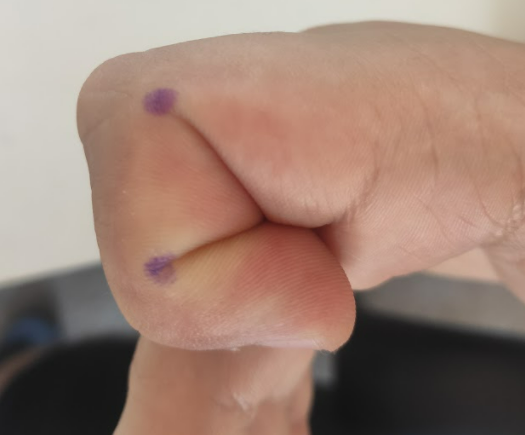

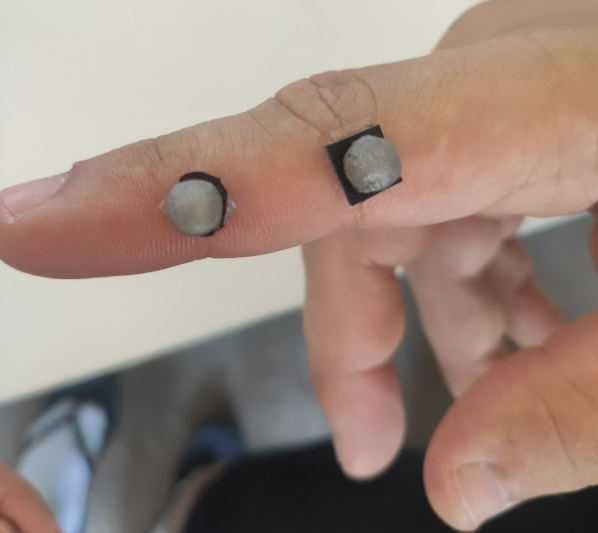

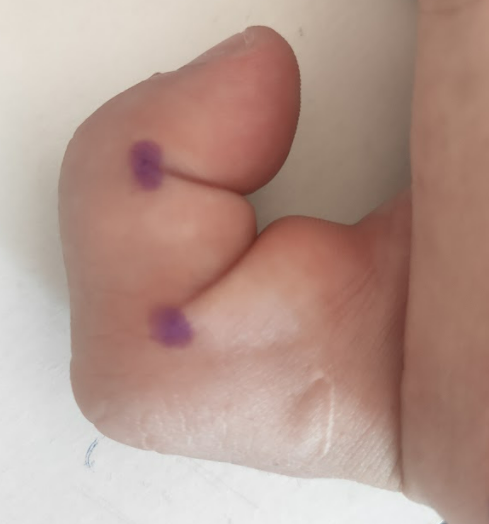

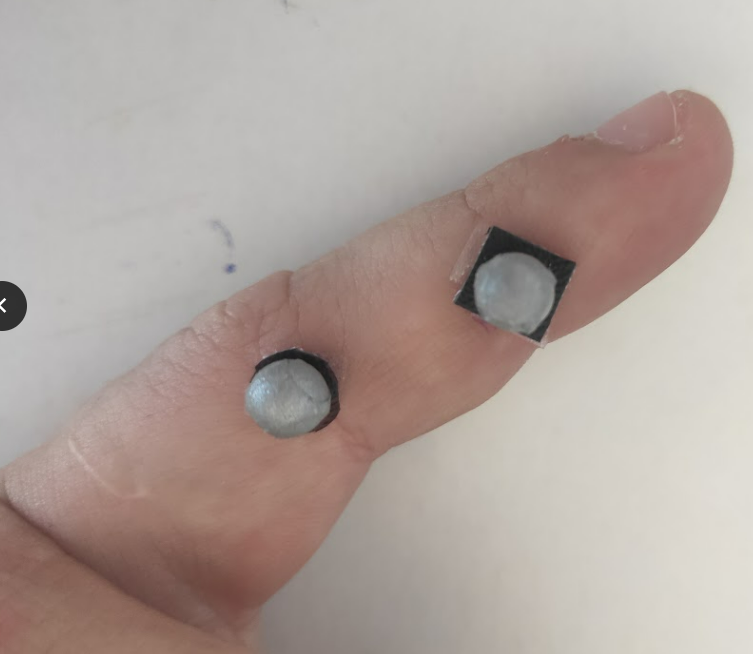


- **#Tip:** One marker in the most distal aspect of the finger.


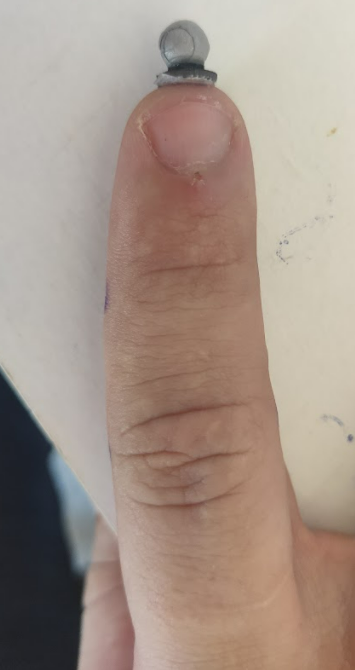


**Tracking markers**

- Index and Middle fingers:

Proximal, middle, and distal phalanges: 3 marker cluster. Dorsal aspect.


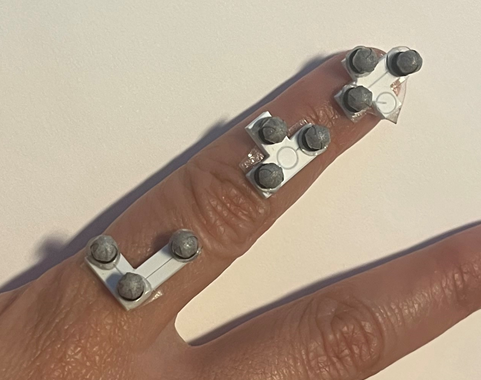


- Ring and little fingers:

Proximal phalanges: 3 marker cluster. Dorsal aspect.

Middle and distal phalanges: 1-2 marker cluster. Dorsal aspect.

All clusters must be aligned using a ruler to ensure there are 2 collinear markers per segment along the finger. The 2 collinear markers per segment should be within the same line of the whole finger.


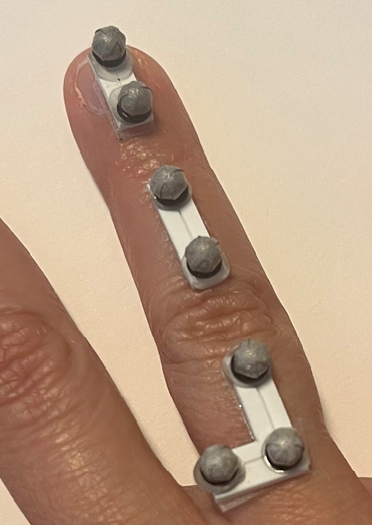


**Thumb segments**

**Calibration markers**

- **Dobj**: using the anatomical snuff box (*Triangular impression on radial, dorsal aspect of the wrist, the scaphoid and trapezium bones form the floor. Extensor pollicis longus is posterior border. Extensor pollicis brevis /abductor pollicis longus are the anterior border. Styloid process of the radius is proximal border.*)

From the head of the 1^st^ metacarpal, moving towards the snuff box, find the base of the metacarpal and position the marker below. (Between metacarpal base and the trapezium) i.e., trapezial articulate surface.


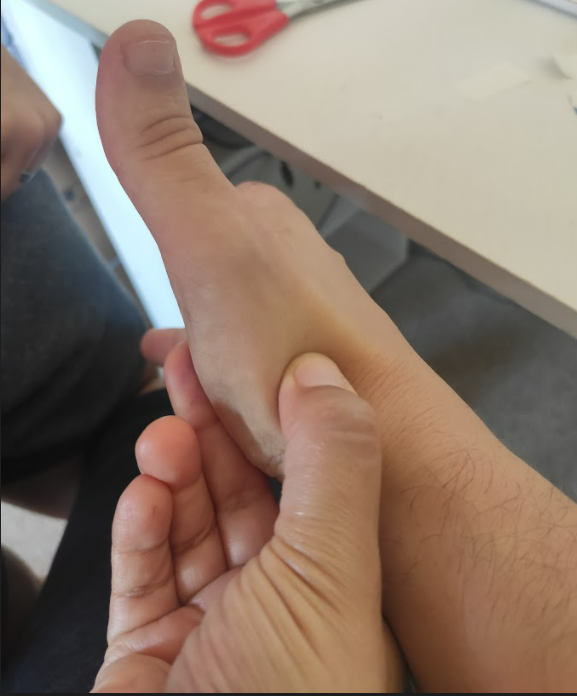

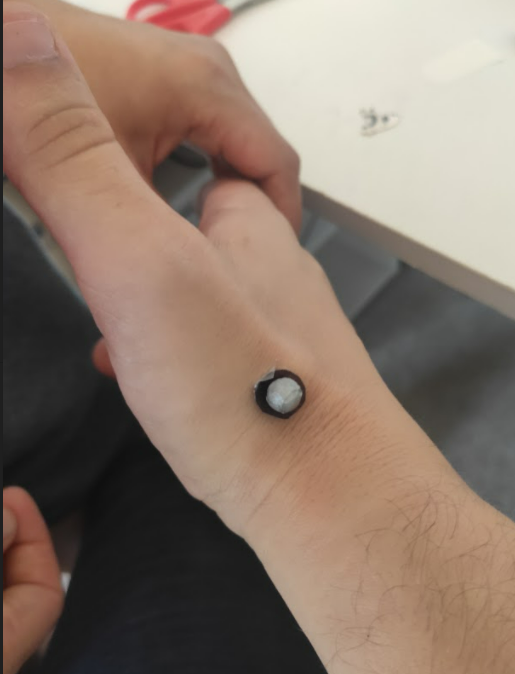


- **Pobj**: On the palmar side, palpate the base of the thumb’s metacarpal bone until a depression where it meets the trapezium bone. It helps to move the thumb while holding on the Dobj point when locating the palmar side of the basal join.


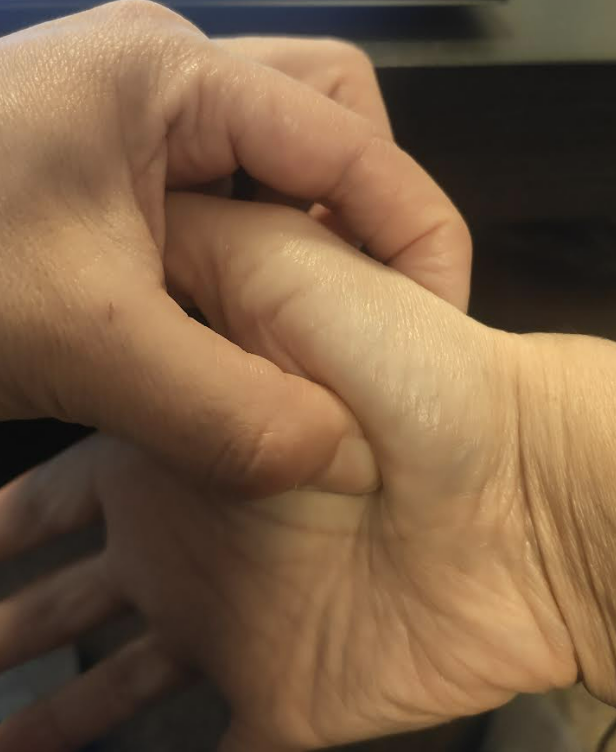


- **Rmcp and Umcp joint markers**.
  - One on each side of the metacarpal head. Rmcp (radial side), Umcp (ulnar side).


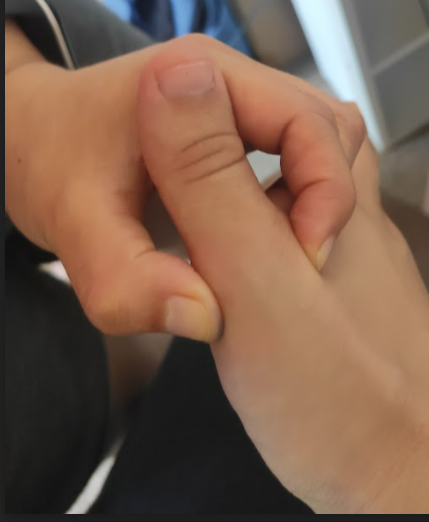

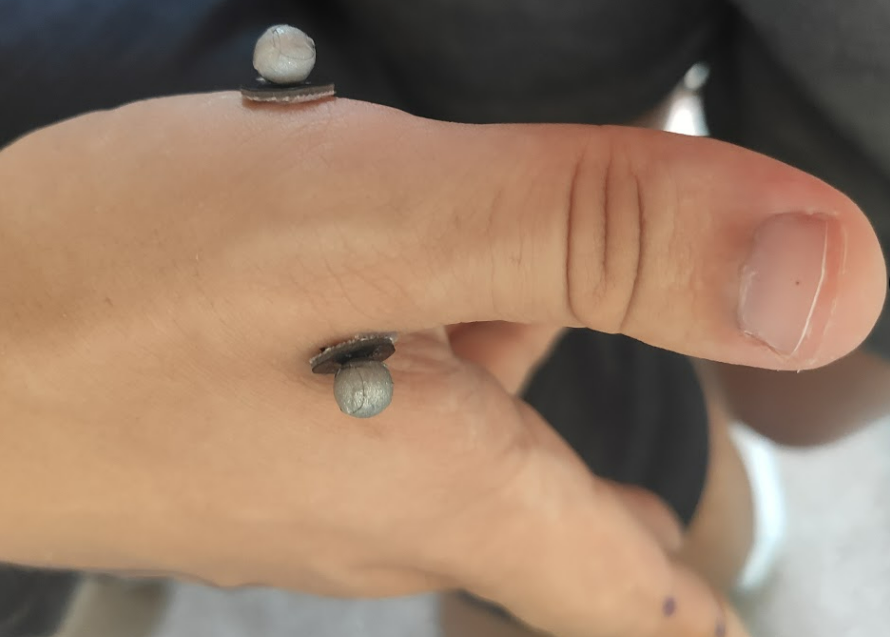


- **Rip:** positioned on the radial condyle of the proximal phalanx
- **Uip:** positioned on the ulnar condyle of the proximal phalanx


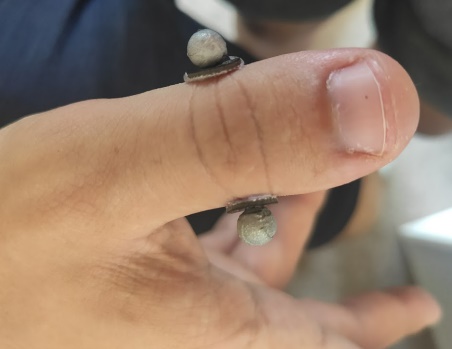


The markers can be adjusted by position them on a virtual dot on top of the crease when the finger is fully flexed.


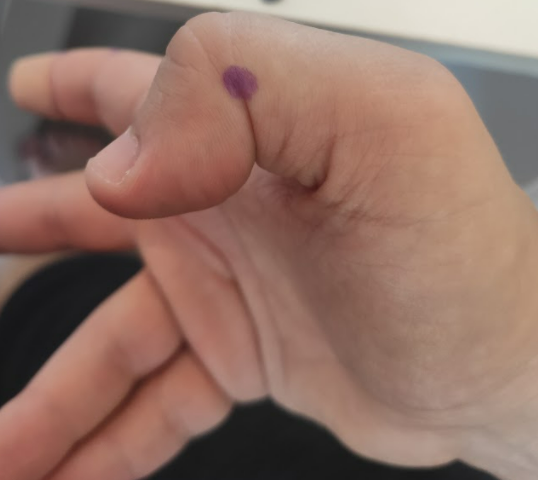

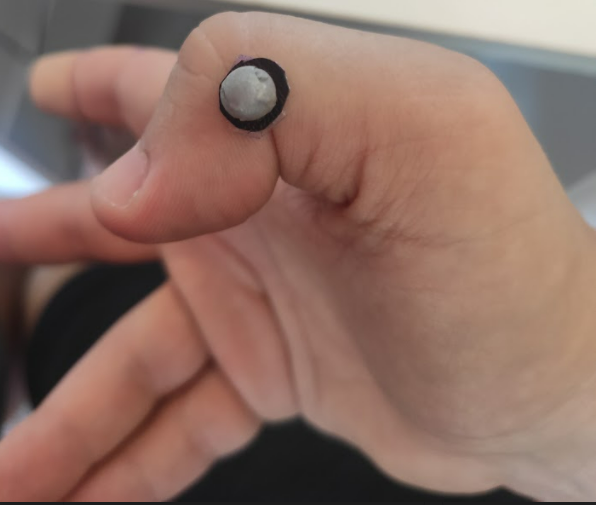

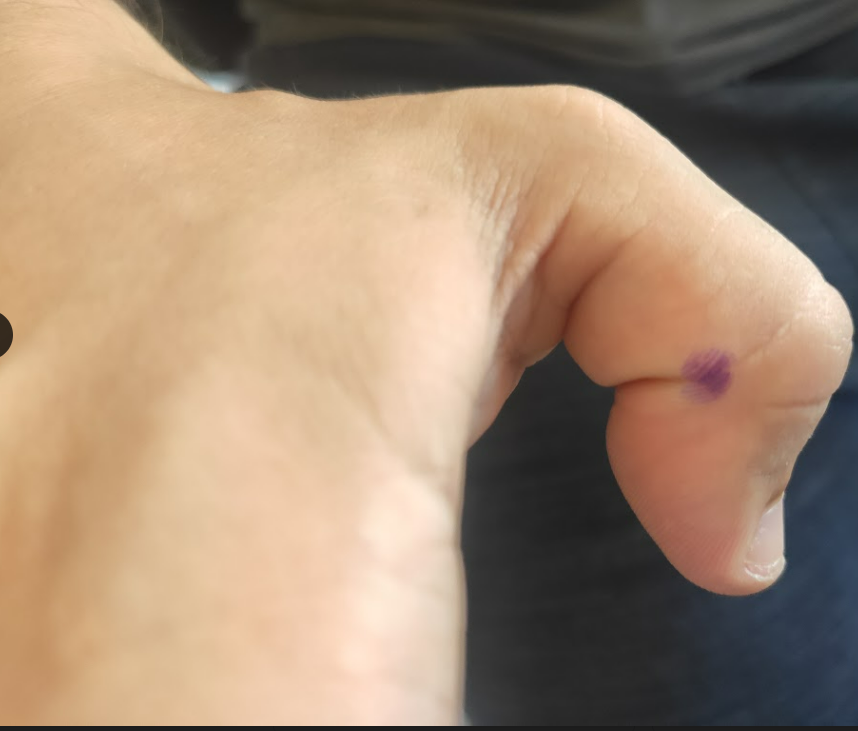

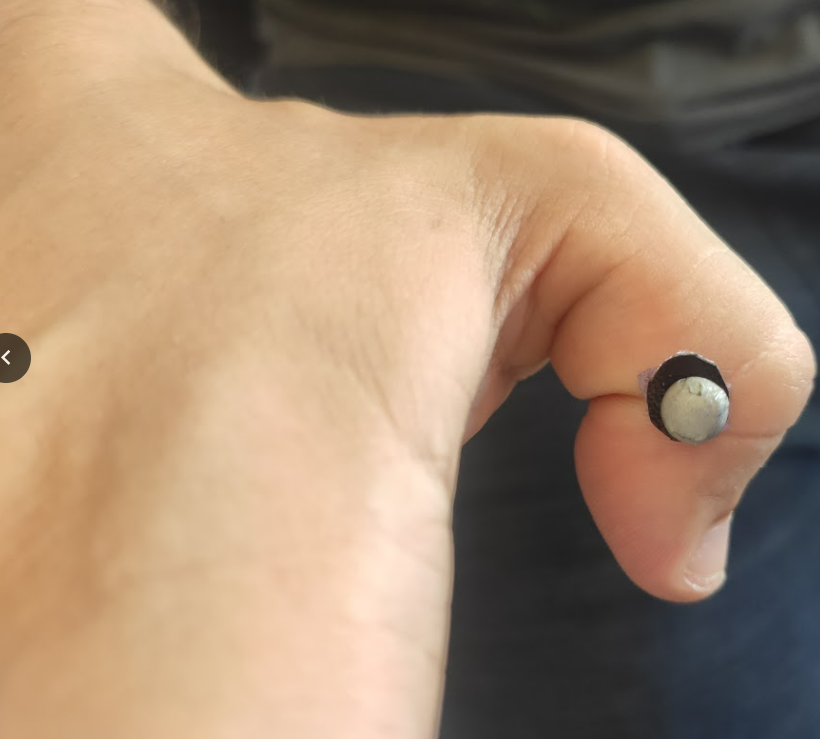


- **1-Tip:** One marker in the most distal aspect of the finger.


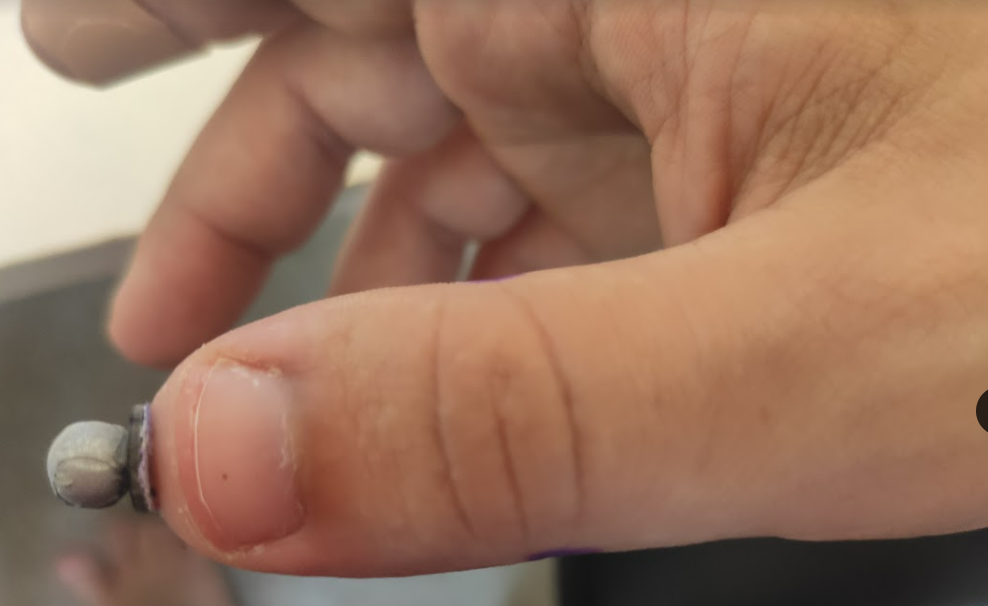


**Tracking markers**

- First metacarpal: 3-4 marker cluster. Dorsal aspect. Proximal phalanx: 3-4 marker cluster. Dorsal aspect. Distal phalanges: 3 marker cluster. Dorsal aspect.


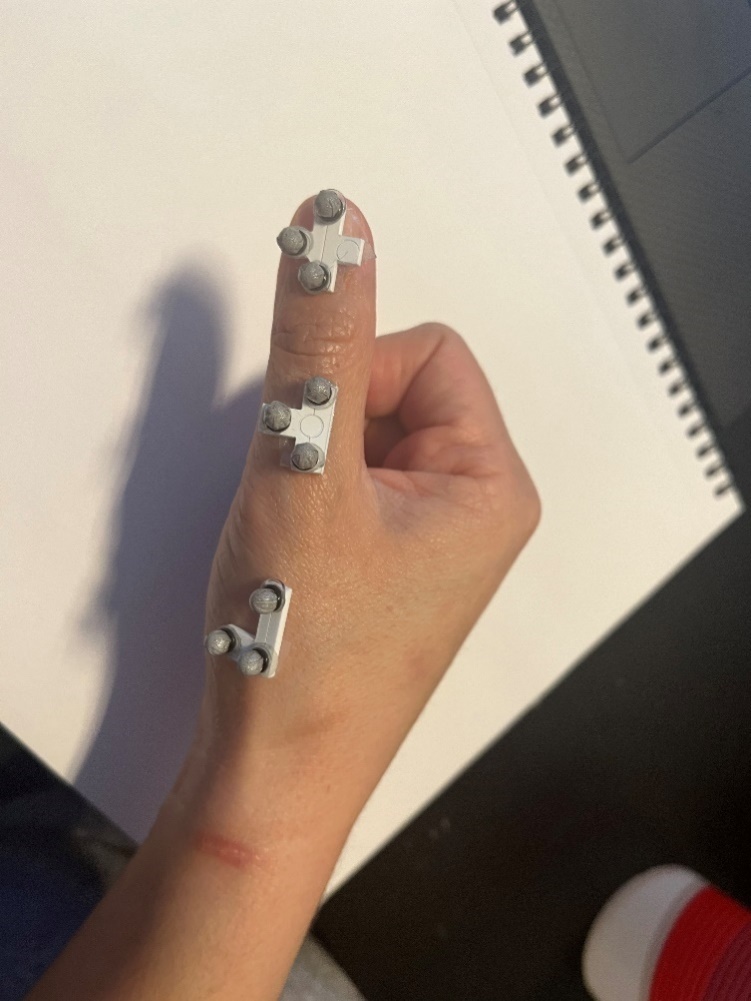

Supplement: Multimedia component 1 [file mmc1.docx]
